# Adverse effects of ethyl esters or oxidation products in omega-3 preparations?

**Published:** 2014-04

**Authors:** Heinz Rupp, Karin G Rupp

**Affiliations:** Experimental Cardiology Laboratory, Department of Internal Medicine – Cardiology, Philipps University, Marburg, Germany; Experimental Cardiology Laboratory, Department of Internal Medicine – Cardiology, Philipps University, Marburg, Germany

## Dear Sir

We read with interest the article by Opperman and Benade, titled Analysis of the omega-3 fatty acid content of South African fish oil supplements: a follow-up study.^1^ We make the following comments.

In humans, long-chain omega-3 fatty acids are required in numerous cellular mechanisms and are converted in small amounts from the plant-based precursor alpha-linolenic acid. It therefore seemed paradoxical when we found that in patients with dilative heart failure, the blood levels of eicosapentaenoic acid (EPA) and docosahexaenoic acid (DHA) were reduced.^2^,^3^ We concluded that a deficiency in highly unsaturated fatty acids (HUFA) represents a key defect contributing to the rapid progression of heart failure requiring replacement therapy.^4^ It is therefore important to assess whether dietary supplement and pharmaceutical-grade omega-3 preparations vary not only in environmental pollutants,^5^ but also in adverse oxidation products.

Opperman and Benade^1^ assessed differences in dietary supplement fish oils available on the South African market. Compared with their 2009 survey, almost a third of the supplements contained ethyl esters (EEs), which appeared to be associated with higher EPA and DHA levels. Although we share their contention that the presence of EEs should be declared on the supplement label, we do not see the evidence for their statement that the safety of a daily intake of EEs has not been confirmed in humans.

In a major prospective, randomised clinical trial by the Gissi-Hf investigators,^6^ EPA/DHA was administered as EEs (omega-3-acid ethyl esters 90, Omacor®) manufactured as a drug according to FDA and EMA regulations. Adverse drug reactions did not differ from the placebo olive oil. Also the rate of discontinuation was not different from a vitamin E supplement.^7^

EEs are split in the gastrointestinal tract. When the intake of EEs and triglycerides was examined in humans, no differences in serum EPA and DHA levels were observed.^8^ EEs can therefore not accumulate in organs, as mentioned by Opperman and Benade.^1^ However, since EPA/DHA in the form of EEs appear to be more prone to autoxidation, the safety of dietary supplements containing EEs needs greater attention.

Did the authors find differences in the levels of peroxides and conjugated dienes depending on the ester form? Did they calculate the intake of peroxides and conjugated dienes associated with a given EPA/DHA intake?

According to our recent study on 63 dietary supplement fish oils from 13 countries,^9^ the peroxide intake for 1 g of EPA + DHA would be 8.6 ± 6.1 times higher compared with omega-3-acid ethyl esters 90. The intake of secondary oxidation products measured as aldehydes would be 10.9 ± 4.4 times higher. In this context it is important to note that the ingestion of less oxidised omega-3 supplements reduced circulating triglyceride and cholesterol levels, as opposed to highly oxidised omega-3 capsules, which had a negative effect on cholesterol levels.^10^

In view of the increasing evidence that the oxidation level of omega-3 fatty acid supplements can be a health risk, we consider it timely to change the perception that EPA/DHA is beneficial, irrespective of the source and the presence of oxidation products. Of particular concern is that the majority of fish oils exhibit a peroxide content above the recommended level of the Global Organisation for EPA and DHA Omega-3 (GOED), i.e. > 80% (South African market, photometric method),^1^ 89% (market of 13 countries, photometric method),9 and 93% (Norwegian market, AOCS official method).^11^
